# Color image encryption scheme based on alternate quantum walk and controlled Rubik’s Cube

**DOI:** 10.1038/s41598-022-18079-x

**Published:** 2022-08-22

**Authors:** Jingbo Zhao, Tian Zhang, Jianwei Jiang, Tong Fang, Hongyang Ma

**Affiliations:** 1 School of Information and Control Engineering, Qingdao University of Technology, Qingdao, 266000 China; 2 School of Science, Qingdao University of Technology, Qingdao, 266000 China

**Keywords:** Quantum information, Information technology

## Abstract

Aiming at solving the trouble that digital image information is easily intercepted and tampered during transmission, we proposed a color image encryption scheme based on alternate quantum random walk and controlled Rubik’s Cube transformation. At the first, the color image is separated into three channels: channel R, channel G and channel B. Besides, a random sequence is generated by alternate quantum walk. Then the six faces of the Rubik’s Cube are decomposed and arranged in a specific order on a two-dimensional plane, and each pixel of the image is randomly mapped to the Rubik’s Cube. The whirling of the Rubik’s Cube is controlled by a random sequence to realize image scrambling and encryption. The scrambled image acquired by Rubik’s Cube whirling and the random sequence received by alternate quantum walk are bitwise-XORed to obtain a single-channel encrypted image. Finally the three-channel image is merged to acquire the final encrypted image. The decryption procedure is the reverse procedure of the encryption procedure. The key space of this scheme is theoretically infinite. After simulation experiments, the information entropy after encryption reaches 7.999, the NPCR is 99.5978%, and the UACI is 33.4317%. The encryption scheme with high robustness and security has a excellent encryption effect which is effective to resist statistical attacks, force attacks, and other differential attacks.

## Introduction

As multimedia technology is growing well today, an increasing number of fields are gradually developing in the direction of digitization and informatization, which has brought convenience to our lives and work. However, the security and confidentiality of data in the process of information transmission are becoming more and more important image plays. As one of the important carriers in the process of information transmission, digital images play a pivotal role in many fields, such as education, finance, medical treatment and so on. However, digital images are easily intercepted and tampered during transmission, which greatly threatens the privacy of image information^1–4^. In view of the security of digital images, many domestic and foreign researchers have brought forward various image encryption methods. For example, digital image encryption schemes are based on chaotic systems^5–8^, which control the placement of image pixels by generating random sequences through the chaotic system. Based on digital image encryption schemes such as Fourier Transform^9,10^, the image is transformed into the frequency domain, and then the amplitude value of the sine and cosine function of each frequency in the frequency domain is operated to realize image encryption; there are also classical digital image encryption methods, such as: Arnold transformation^11–14^, AES transformation^15–18^, DNA encoding encryption^19–25^, etc. Based on the alternate quantum random walk and controlled Rubik’s Cube transform, this paper comes up with a novel digital image encryption scheme. And the encryption algorithm designed by him makes full use of the characteristics of quantum random walk, which has the advantages of large key space and key sensitivity.

With the continuous development of quantum computing and quantum communication, many quantum algorithms and quantum technologies have emerged^26–34^. Quantum random walk is generated by applying classical random walk to quantum computing, and it plays an important part in a number of quantum algorithms^35–38^. Compared with classical random walking, quantum random walk has two main advantages, one is fast running speed, and the other is strong security. Similar to chaotic systems, quantum random walk has many excellent properties: sensitivity to initial values, stability, non-periodicity, etc. Thence, the key space of quantum random walk is very vast, and it has a perfect capability to resist external malicious attacks. Therefore, quantum random walk is very advantageous in the field of image encryption. Wang et al. designed an image encryption algorithm that combines quantum random walk with DNA encoding^19^. Based on quantum random walk and double random phase encoding technology, Abd-El-Atty et al. put forward an image encryption scheme^39^. Based on quantum walk Abd-El-Atty et al. conducted in-depth research and proposed multiple algorithms for image encryption combined with classical algorithms^45–47^. MA et al. designed an image encryption scheme that combines alternating quantum random walk with discrete cosine transform, which makes full use of the characteristics of quantum random walk, which has the advantages of large key space and key sensitivity.

The principle of Rubik’s Cube transformation is inspired by Rubik’s Cube. The Rubik’s Cube is to change the position of the sub-block by moving the sub-blocks, so as to realize the scrambling of the Rubik’s Cube. Similarly, applying the Rubik’s Cube transformation to image scrambling is to scramble the image by moving the position of the image pixels^40–44^. For a third-order Rubik’s Cube, if a certain layer is rotated 90 degrees at a time, there are eighteen ways for rotation. There are several ways of permutation and combination of a 3rd-order Rubik’s Cube, but it is the only way to restore, so the computational complexity is very high. Thus it is feasible to combine the Rubik’s Cube transformation with the image encryption. Zhang et al. proposed an image encryption scheme based on Rubik’s Cube transformation and chaotic sequence^41^. Loukhaoukha et al. designed an image encryption method based on the Rubik’s Cube rotation principle^43^, and using the principle of Rubik’s cube rotation, this encryption algorithm can scramble and encrypt the image very well, but its key space is small. At first, the original image was scrambled by using the Rubik’s Cube principle, and then the rows and columns of the scrambled image were XORed with the key. Vidhya et al. designed a chaotic image encryption algorithm based on Rubik’s Cube transformation and prime number decomposition algorithm^44^, and the proposed method makes full use of the Rubik’s cube principle to achieve bit-level image encryption, and has a good scrambling effect. I think that if we can add pixel space scrambling, it will achieve a better encryption effect.

This article combines quantum random walk with Rubik’s Cube transformation to complete the encryption of digital images. Firstly, a random sequence is generated by quantum walking. Then the random sequence is used to control the magic cube transform to achieve the purpose of image scrambling. The full text of this article is structured as follows: The second part introduces the relevant knowledge needed in the paper. Next part introduces the principles and processes of image encryption and decryption. The Fourth part presents the simulation results and the analysis of the simulation results. Finally, we draw a conclusion about the scheme.

## Principle

### Alternate quantum walks

Quantum walk can be separated into two parts: One is discrete time quantum random walk and the other is continuous time quantum random walk. We focus on the former way of random walking in this paper.

Similar to the classic random walk, quantum walk is mainly composed of coin register (coin space ${\mathcal {H}}^{c}$) and rambler location information (rambler’s location space ${\mathcal {H}}^{l}$).Therefore, the quantum walk is carried out in Hilbert space ${\mathcal {H}}={\mathcal {H}}^{C} \otimes {\mathcal {H}}^{P}$. The process of quantum random walk is separated into two steps. The first step is to apply the coin operator on the coin state of the two-dimensional Hilbert space ${\mathcal {H}}^{c}$, and then apply the unitary operator ${\hat{U}}$ to the total Hilbert space ${\mathcal {H}}$. Thus the quantum also can be seen as the application of a unitary operator U that acts repeatedly on the quantum walk system and the operator can be descried as:

1 $$\begin{aligned} {\hat{U}}=S C=S({\hat{C}} \otimes I) \end{aligned}$$

Assume that the quantum walk coin operator always chooses the same operator${\hat{C}}$:

2 $$\begin{aligned} {\hat{C}}=\left( \begin{array}{cc}\cos \alpha &{} \sin \alpha \\ -\sin \alpha &{} \cos \alpha \end{array}\right) \end{aligned}$$

when $\alpha =\frac{\pi }{4}$. the coin operator can be expressed as:

3 $$\begin{aligned} {\hat{C}}=\frac{\sqrt{2}}{2}\left( \begin{array}{ll}1 &{} 1 \\ -1 &{} 1\end{array}\right) =H \end{aligned}$$

The transfer operator *S* is used in quantum walks to manipulate the walker to decide the direction of the next walk. When the condition of coin state is $|0\rangle$ (spin up$|\uparrow \rangle$), the walker will move forward in a certain direction. While the condition of coin state is $|1\rangle$ (spin down$|\downarrow \rangle$), the walker will take one step further in the opposite direction. So the transfer operator ${\mathcal {S}}$ can be denoted as:

4 $$\begin{aligned} S=|0\rangle \left\langle 0\left| \otimes \right| n+1\right\rangle \langle n|+| 1\rangle \left\langle 1\left| \otimes \right| n-1\right\rangle \langle n| \end{aligned}$$

In the alternate quantum walk, it is formed by the position state tensor$\{|{\mathbf {x}}, {\mathbf {y}}\rangle , x, y \in Z\}$, walking alternately in two directions in a two-dimensional space. Therefore, in the quantum random walk process, the unitary operator repeatedly acting on the quantum walk system can be denoted as:

5 $$\begin{aligned} {\hat{U}}= & {} {\hat{S}}_{y}(I \otimes {\hat{C}}) {\hat{S}}_{x}(I \otimes {\bar{C}})={\hat{S}}_{y}(I \otimes H) {\hat{S}}_{x}(I \otimes H)\end{aligned}$$

6 $$\begin{aligned} {{{\hat{S}}}_{y}}= & {} |0\rangle \langle 0|\otimes \sum \limits _{m,n\in Z}{|n+1,m\rangle \langle n,m|}\nonumber \\&+|1\rangle \langle 1|\otimes \sum \limits _{m,n\in Z}{|n-1,m\rangle \langle n,m|}\end{aligned}$$

7 $$\begin{aligned} {{{\hat{S}}}_{x}}= & {} |0\rangle \langle 0|\otimes \sum \limits _{m,n\in Z}{|n,m+1\rangle \langle n,m|}\nonumber \\&+|1\rangle \langle 1|\otimes \sum \limits _{m,n\in Z}{|n,m-1\rangle \langle n,m|} \end{aligned}$$

**Figure 1:**
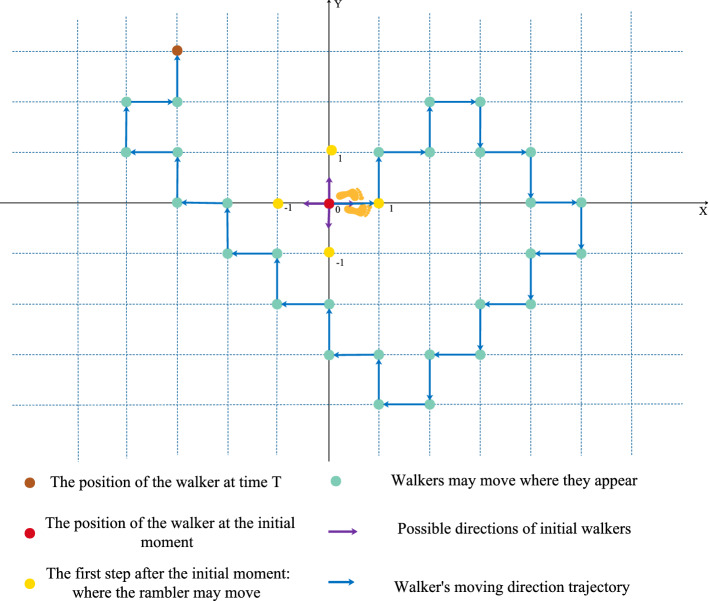
Alternate quantum walk.

When the coin state is $|0\rangle$ ($|1\rangle$), act on the walker to walk up (down) along the Y axis , and act on the walker to make it walk right (to the left) along the X axis as shown in Fig. 1. Assuming that the walker is locally at the initial moment $(\mathrm {y}, x)=(0,0)$, the initial state of the coin state is a superposition state $|{\text {coin}}\rangle =a|0\rangle +b|1\rangle$, and the quantum state of the initial quantum walk can be expressed as:$\left| \psi _{0}\right\rangle =|00\rangle \otimes \mid$ coin $\rangle$. Here, after walking N steps, the final quantum state of the entire system is$\left| \psi _{N}\right\rangle ={\hat{U}}^{N}\left| \psi _{0}\right\rangle$, The probability that the walker is at the location$(\mathrm {y}, x)$ is:

8 $$\begin{aligned} P_{Y,X}=\sum \left| \left\langle y, x, 0\left| {\hat{U}}^{N}\right| \psi _{0}\right\rangle \right| ^{2}+\sum \left| \left\langle y, x, 1\left| {\hat{U}}^{N}\right| \psi _{0}\right\rangle \right| ^{2} \end{aligned}$$

### Rubik’s Cube transform

The concept of Rubik’s Cube transformation comes from Rubik’s Cube toys, which disrupt the patterns on the surface of the Rubik’s Cube by rotating the cubes. The algorithm in this paper is based on the third-order Rubik’s Cube. The third-order Rubik’s Cube is a special cube that is composed of 26 sub-blocks and can be rotated along each axis. The six faces of the Rubik’s Cube have different colors.

**Figure 2:**
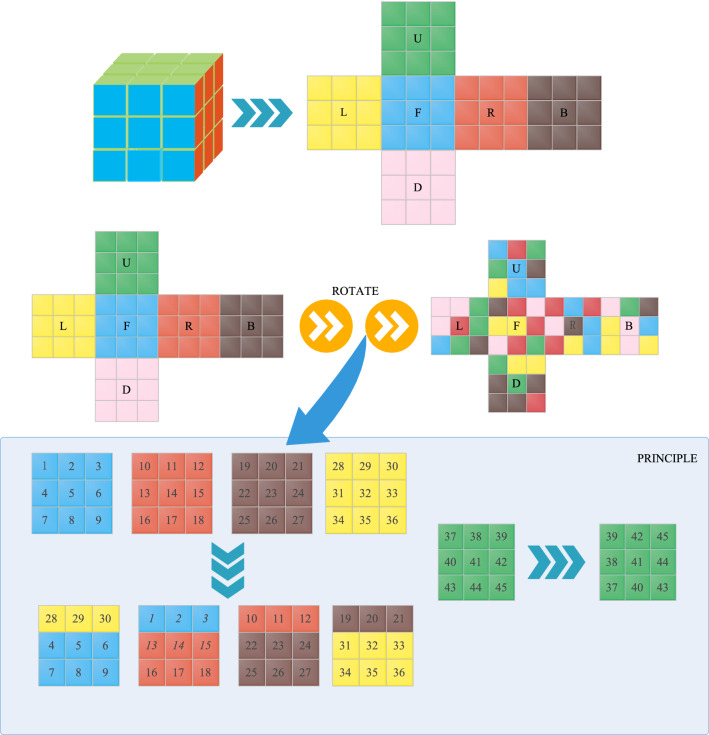
Third-order Rubik’s Cube principle. The upper part is the expansion diagram of the Rubik’s Cube, the middle is the Rubik’s Cube rotation, and the lower layer is the basic Rubik’s Cube rotation principle.

For a 3rd-order Rubik’s Cube, we firstly determine the representation of the six faces of the Rubik’s Cube, and mark each sub-block of the Rubik’s Cube. Third-order Rubik’s Cube expansion map is displayed in Fig. 2, the top side is represented as U, the front side is represented as F, the right side is represented as R, the bottom side is represented as D, the back side is represented as B, and the left side is represented as L. Because U surface and D surface, R surface and L surface, and F surface and B surface are relative, we only consider the three surfaces: U, R, and F. For example, when the first layer of the U side of the Rubik’s Cube is rotated 90° to the right, the state of the Rubik’s Cube is demonstrated in Fig. 2. And the U surface is rotated 90° counterclockwise, while the D surface does not change. When the middle layer of the U side of the Rubik’s Cube is rotated 90 degrees, the middle layers of the four sides of F, R, B, and L are also cyclically shifted, while the U and D surfaces do not change. Similarly, the same principle applies to rotating other surfaces.

Through the above principles, rotating the Rubik’s Cube can be pieced together into a specific pattern, or the specific pattern can be messed up. We apply the theory of Rubik’s Cube transformation to image encryption. The pixels of the image are mapped to the Rubik’s Cube, and a sub-block of the Rubik’s Cube is regarded as a pixel on the image. According to the principle of Rubik’s Cube transformation and a specific whirling rule, the pixel position of the original image is shuffled to generate an irregular image. The recipient can use the key to decrypt the encrypted image to acquire the original image. Therefore, the privacy and security of image information in the transmission process can be improved.

## Principle of encryption and decryption

In this paper, a random probability matrix is generated by alternating quantum walks and transformed an one-dimensional sequence,the rotation of the Rubik’s Cube is controlled by this sequence. Through rotation, the scrambling image is XORed with the matrix converted from the random probability matrix to obtain the encrypted image.

**Figure 3:**
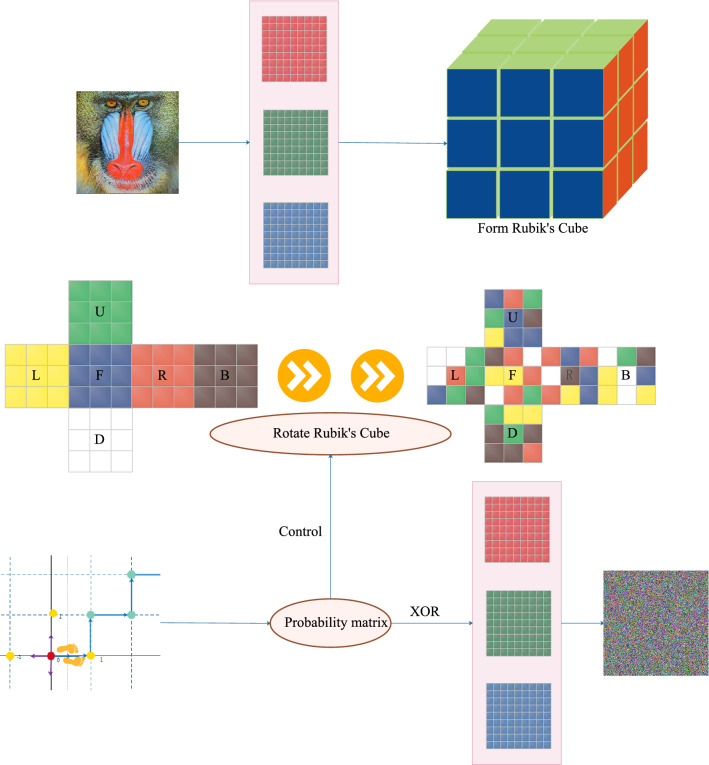
Encryption flowchart. Separate the three channels, select pixels to form a Rubik’s Cube, use alternate quantum walks to control the Rubik’s cube rotation, scramble and encrypt, merge the three channels to get an encrypted image.

### Encryption algorithm

The random sequence can be obtained through quantum walk, which makes the image difficult to be eavesdropped on. Therefore the scheme has good security. The detailed steps of the encryption algorithm are as follows (Fig. 3 is the encryption flowchart):

- Step 1: Enter the image ${\mathcal {I}}$ to be encrypted, analyze the image information, especially the size information${\mathcal {I}}(\mathrm {m}, \mathrm {n}, 3)$. Split the original color image ${\mathcal {I}}(\mathrm {m}, \mathrm {n}, 3)$ into ${\mathcal {R}}(\mathrm {m}, \mathrm {n})$, ${\mathcal {G}}(\mathrm {m}, \mathrm {n})$, ${\mathcal {B}}(\mathrm {m}, \mathrm {n})$ three-channel images and represent them in a pixel matrix:
  9 $$\begin{aligned} {\mathcal {I}}(\mathrm {m}, \mathrm {n}, 3)={[{\mathcal {R}}(\mathrm {m}, \mathrm {n}),{\mathcal {G}}(\mathrm {m}, \mathrm {n}),{\mathcal {B}}(\mathrm {m}, \mathrm {n})]} \end{aligned}$$
- Step 2: Select the parameters $\left( N_{1}, N_{2}, a, b\right)$ of the alternate quantum walk, walk ${\mathcal {N}}$ steps on the initial state $\psi _{0}$, and generate a probability distribution matrix: $P_{y, x}$ of size$(\mathrm {m}, \mathrm {n})$:
  10 $$\begin{aligned} P_{Y,X}=\sum \left| \left\langle y, x, 0\left| {\hat{U}}^{N}\right| \psi _{0}\right\rangle \right| ^{2}+\sum \left| \left\langle y, x, 1\left| {\hat{U}}^{N}\right| \psi _{0}\right\rangle \right| ^{2} \end{aligned}$$
- Step 3: Divide the single-channel image into 6 parts without superimposition to obtain 6 sub-images of different matrices, and treat the 6 matrices as 6 faces of the Rubik’s Cube-front (F), back (B), and top (U) , Bottom surface (D), left side (L), right side (R):
  11 $$\begin{aligned} I=(F,B,U,D,L,R) \end{aligned}$$
- Step 4: Take out the $3*3$ pixel matrix from the six matrices to form six faces and form a 3rd-order cube cube, and the surface has 54 pixel values, so the image can produce a cube.
- Step 5: Obtain the random probability matrix $P_{y, x}$ through the discrete time alternate quantum walk, and convert it to an integer value of [0–17] and use it to represent the rotation method as shown in Table 1:
  12 $$\begin{aligned} K=f i x\left( P_{y, x} \times 10^{16}\right) \bmod 18 \end{aligned}$$
- Step 6: Rotating the Rubik’s Cube, dividing the sequence obtained in discrete time into 6 parts, each part corresponds to a different Rubik’s cube rotation mode, and the set K of integer value is [0–17] representing 18 rotation modes ${\mathcal {R}}_{ot}$.
- Step 7: Rotate each face element of the Rubik’s cube that has just been rotated firstly by row and bitwise right circularly shifted by the value of K, and then circularly shifted bitwise right by column by the value of K:
  13 $$\begin{aligned} K=f i x\left( P_{y, x} \times 10^{16}\right) \bmod 18 \end{aligned}$$
- Step 8: Convert the random matrix $P_{y, x}$ to an integer matrix of [0–255]: $L=f i x\left( P_{y, x} \times 10^{16}\right) \bmod 256$, and then react to the third step, and perform bitwise XOR processing with rotated matrix to obtain a single-channel encrypted image.
  14 $$\begin{aligned} {{I}_{en}}= {{I}_{1}} \oplus L={{I}_{1}}\oplus [fix({{P}_{y,x}}\times {{10}^{16}})\,\bmod \,256] \end{aligned}$$
- Step 9: Perform the same steps above for three channels, combine the encrypted three channel image of R, G, and B to obtain a color encrypted image.

**Table 1:** Third-order Rubik’s Cube rotation.

Method	Direction	Affected four faces	Surface affected by rotation
U1	Clockwise	F R B L	U
U2	Clockwise	F R B L	NULL
U3	Clockwise	F R B L	D
L1	Clockwise	F D B U	L
L2	Clockwise	F D B U	NULL
L3	Clockwise	F D B U	R
F	Clockwise	U R B L	F
F2	Clockwise	U R B L	NULL
F3	Clockwise	U R B L	B
U1’	Counterclockwise	F R B L	U
U2’	Counterclockwise	F R B L	NULL
U3’	Counterclockwise	F R B L	D
L1’	Counterclockwise	F D B U	L
L2’	Counterclockwise	F D B U	NULL
L3’	Counterclockwise	F D B U	R
F1’	Counterclockwise	U R B L	F
F2’	Counterclockwise	U R B L	NULL
F3’	Counterclockwise	U R B L	B

U1: Rotate the first layer from top to bottom. U2: Rotate the second layer from top to bottom. U3: Rotate the third layer from top to bottom. L1: Rotate the first layer from left to right. L2: Rotate the second layer from left to right. L3: Rotate the third layer from left to right. F1: Rotate the first layer from front to back. F2: Rotate the second layer from front to back. F3’: Rotate the third layer from front to back. U1’: Rotate the first layer from top to bottom. U2’: Rotate the second layer from top to bottom. U3’: Rotate the third layer from top to bottom. L1’: Rotate the first layer from left to right. L2’: Rotate the second layer from left to right. L3’: Rotate the third layer from left to right. F1’: Rotate the first layer from front to back. F2’: Rotate the second layer from front to back. F3’: Rotate the third layer from front to back.

### Decryption algorithm

Decryption is the contrary procedure of encryption. Briefly describe the decryption process.

- Step 1: Split the encrypted color image into R, G, and B three-channel images to obtain three single-channel encrypted images.
- Step 2: Use the parameters selected in the encryption process to perform a discrete time alternate quantum walk, generate a matrix, convert it into a pixel value matrix of [0–255], and take bitwise XOR with the encrypted image
- Step 3: Divide the image matrix into six parts and the sequence obtained in discrete time is divided into six parts.
- Step 4: Convert the probability matrix $P_{y, x}$ to the integer sequence value of [0–17], and perform the Rubik’s Cube reduction based on this.
  15 $$\begin{aligned} \left\{ \begin{array}{l}k^{\prime }=k+3, k \in (0,1,2,6,7,8,12,13,14) \\ k^{\prime }=k-3, k \in (3,4,5,9,10,11,15,16,17)\end{array}\right. \end{aligned}$$
- Step 5: Apply step 3 in the reverse direction, merge the sub-images into a single-channel image of size, and then merge the decrypted three channel image to obtain the original image.

## Experiments and performance analysis

So as to prove that the proposed encryption scheme has sufficient security, we select four color images with size of 512 × 512 for simulation analysis. This section analyzes the histogram, correlation, information entropy, key space, key sensitivity, and PSNR of encrypted images. The parameters of the alternate quantum walk generation key are (512, 512, $\alpha$, $\beta$), $\alpha$, $\beta$ are obtained by calculating the image’s hash.

### Encryption effect

**Figure 4:**
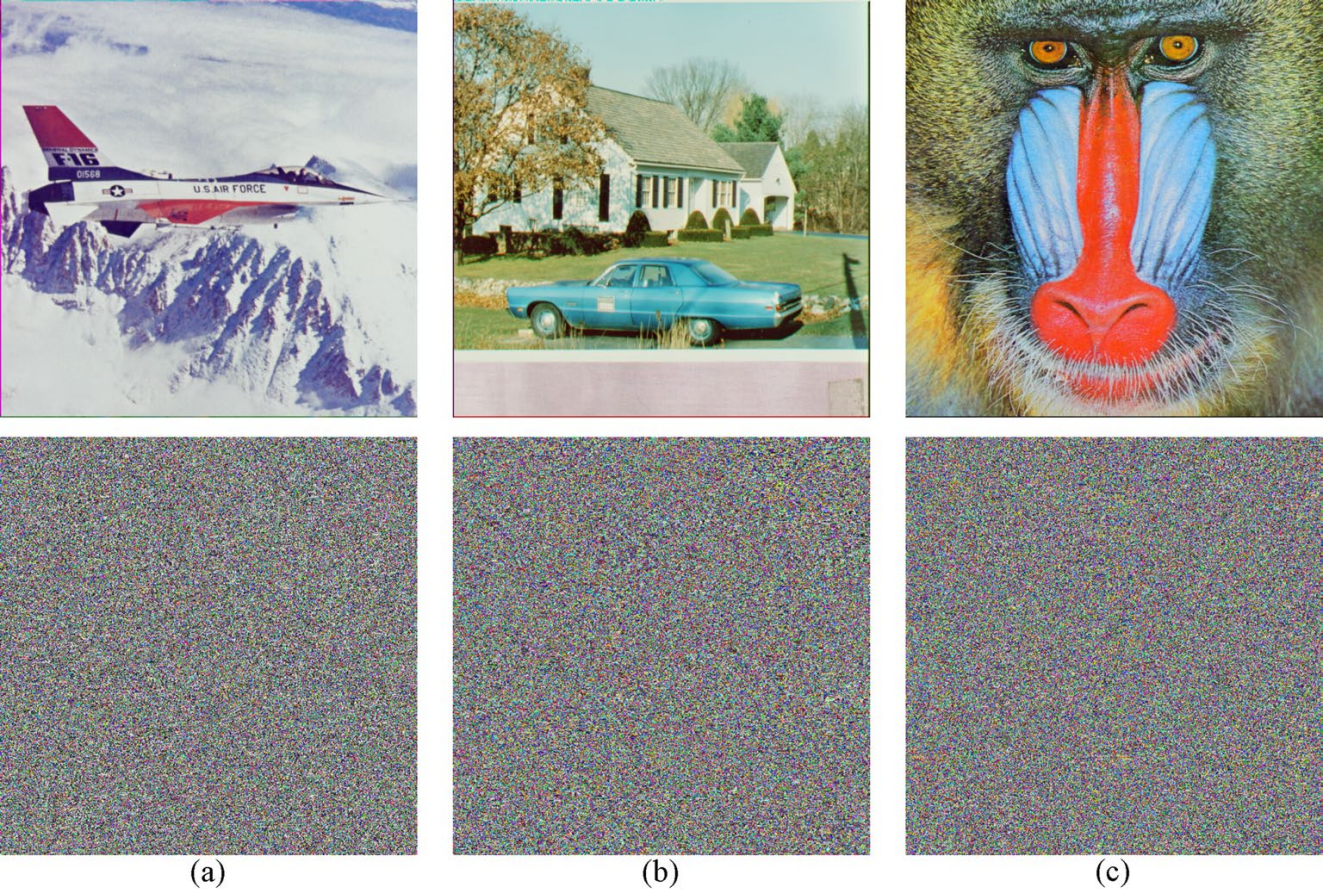
Entryption results. (**a**) Jetplane and it’s encryption image. (**b**) House and it’s encryption image. (**c**) Baboon and it’s encryption image.

We choose three color images to perform a simulation, and the results are demonstrated in Fig. 4. From the Fig. 4, it’s obvious that the encrypted image has no visual information about original image.

### Histogram analysis

**Figure 5:**
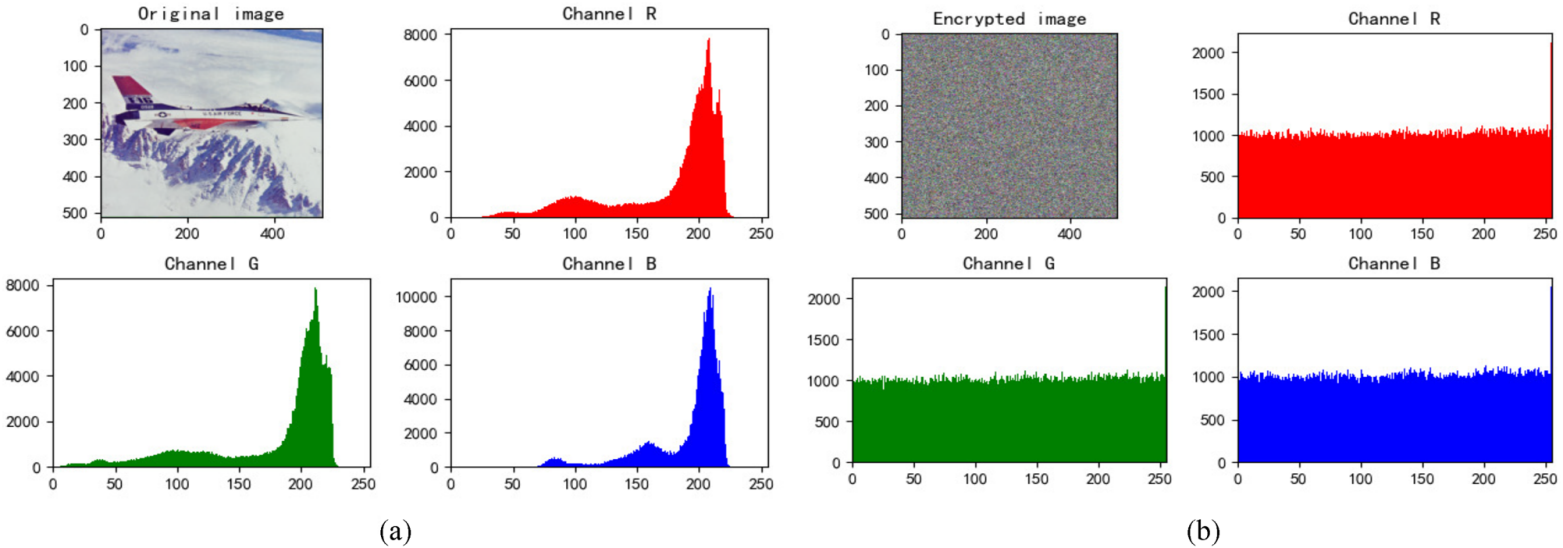
Jetplane’s histogram. (**a**) Histogram of original Jetplane. (**b**) Histogram of encrypted Jetplane.

**Figure 6:**
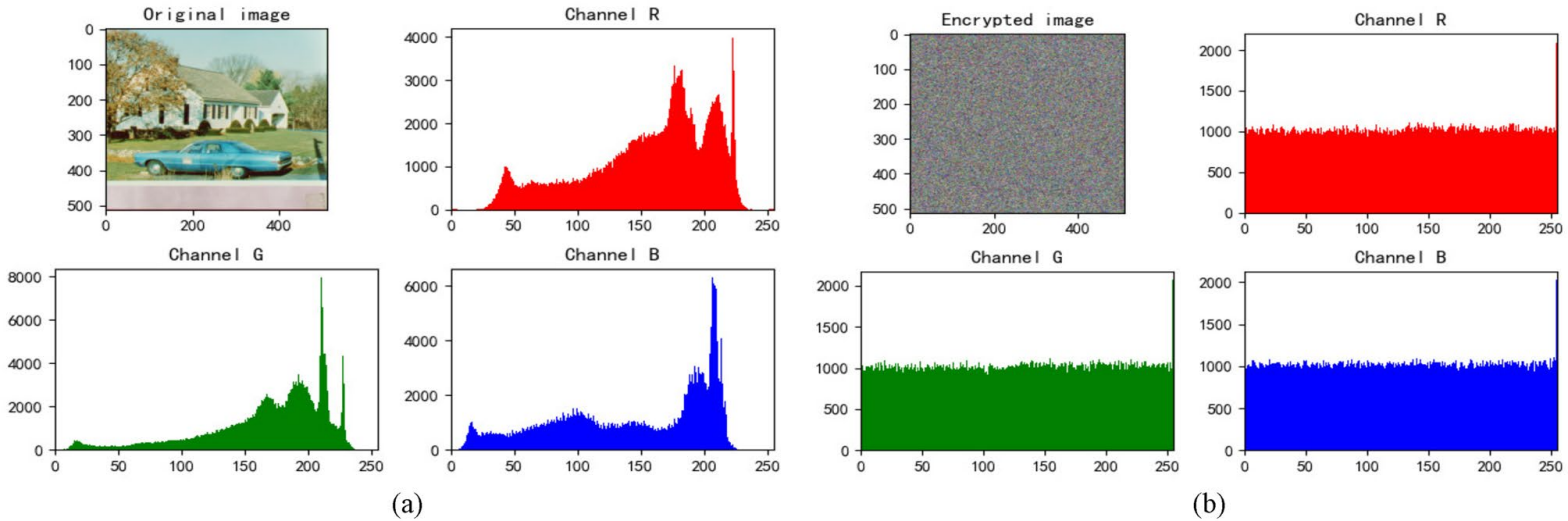
House’s histogram. (**a**) Histogram of original House. (**b**) Histogram of encrypted House.

**Figure 7:**
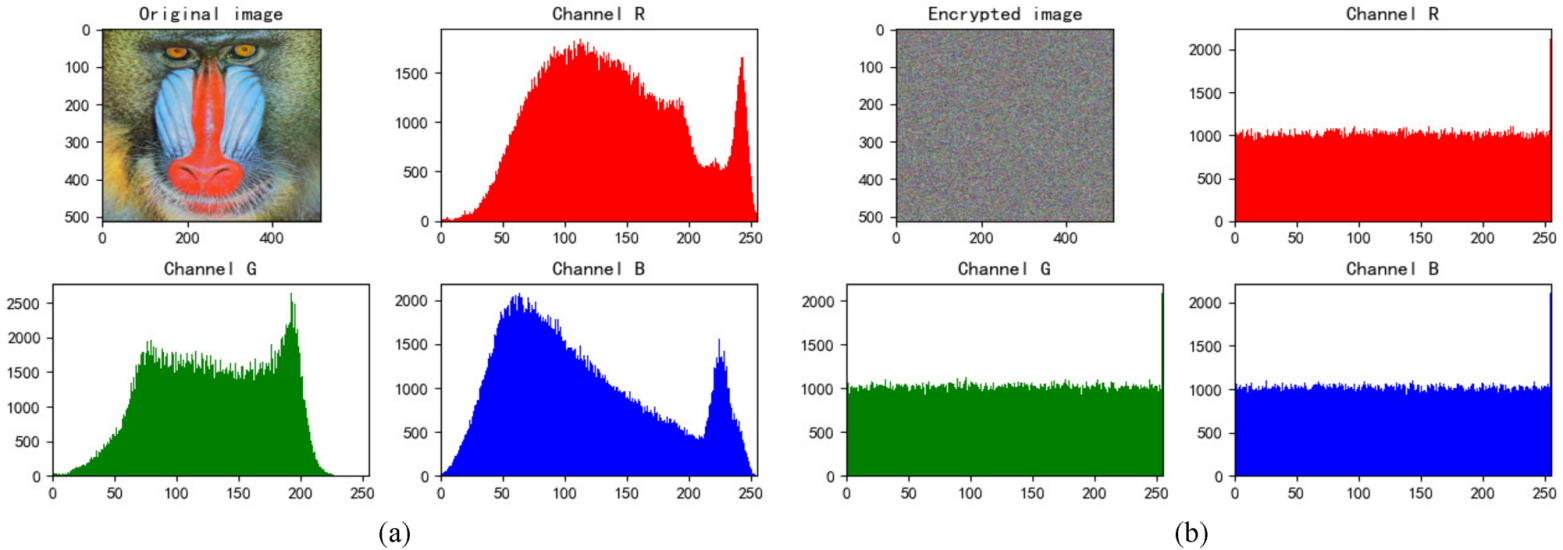
Baboon’s histogram. (**a**) Histogram of original Baboon. (**b**) Histogram of encrypted Baboon.

**Figure 8:**
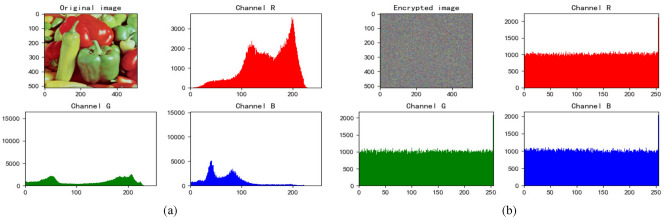
Peppers’s histogram. (**a**) Histogram of original Peppers. (**b**) Histogram of encrypted Peppers.

From the perspective of ciphertext histogram, they tend to be uniform, balance the frequency of each pixel value, and have the capability to resist statistical attacks. The histogram of the original image. The ciphertext is demonstrated in Figs. 5, 6, 7 and 8.

We find that the pixels of the original image are not uniformly distributed, which is easy to be attacked by statistical analysis. The pixel values of encrypted images are evenly dispersed, which can resist statistical analysis attacks well and ensure the security of information.

### Information entrop analysis

Information entropy is usually used to measure the randomness of a system. In the field of image encryption, information entropy is advantaged to weigh the uncertainty of image information. The more evenly the pixel points of each gray level of the encrypted image R, G, and B are distributed, the better the encryption effect and the stronger the capability to resist external attacks. The formula for calculating information entropy is as follows:

16 $$\begin{aligned} H(\alpha )=-\sum _{i=1}^{L} P\left( \alpha _{i}\right) \log _{2} P\left( \alpha _{i}\right) \end{aligned}$$

where $H(\alpha )$ denotes the value of information entropy. The closer its value is to 8, the better the encryption effect is. $\alpha _{i}$ represents the gray value of the first pixel, and$P\left( \alpha _{i}\right)$ represents the probability of the gray level. Measure the entropy of the three images of Lena, House, Baboon and Peppers after encryption, and the results are illustrated in Table 2. Taking Lena image as an example, comparing the encryption method in this paper with the different encryption methods, the results are demonstrated in Table 3, which proves the superiority and the security of the encryption method in this paper. The entropy value of the algorithm which put forward in this paper can reach 7.999, which is better than most encryption algorithms.

**Table 2:** Global and local information entrop of three channels.

Global	Jetplane	House	Baboon	Peppers
Channel R	7.9991	7.9992	7.9992	7.9992
Channel G	7.9991	7.9992	7.9993	7.9993
Channel B	7.9989	7.9994	7.9994	7.9994

Local	Jetplane	House	Baboon	Peppers
Channel R	7.8912	7.8985	7.8947	7.9002
Channel G	7.8986	7.8972	7.8847	7.9000
Channel B	7.9046	7.9005	7.9002	7.9040

**Table 3:** Information entrop of different encryption.

Information entrop	Proposed	Ref^29^	Ref^46^	Ref^16^	Ref^48^	Ref^49^
Jetplane	7.999	******	******	******	******	7.9971
House	7.9992	******	7.99704	7.9969	******	******
Baboon	7.9993	7.9974	7.99729	******	7.9993	7.9995
Peppers	7.9992	******	******	7.9851	7.9993	7.9989

* means no value.

### Correlation analysis

**Figure 9:**
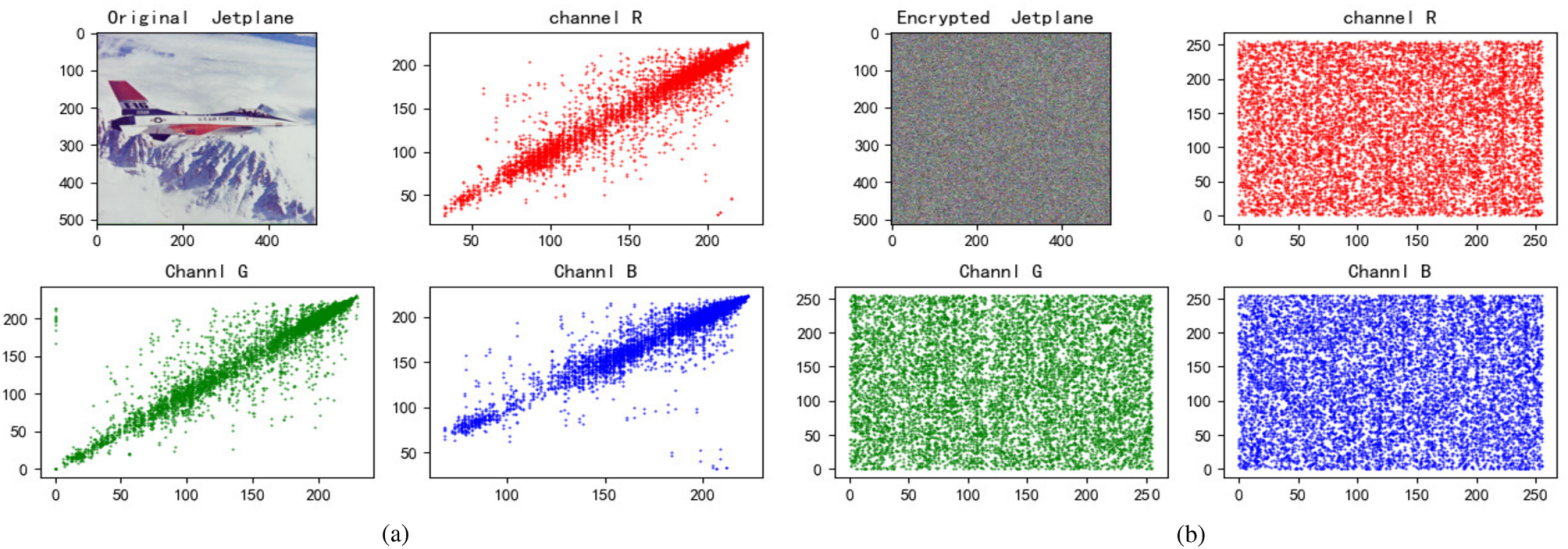
Jetplane’s correlation. (**a**) Correlation of Jetplane and it’s channels. (**b**) Correlation of encrypted Jetplane and it’s channels.

**Figure 10:**
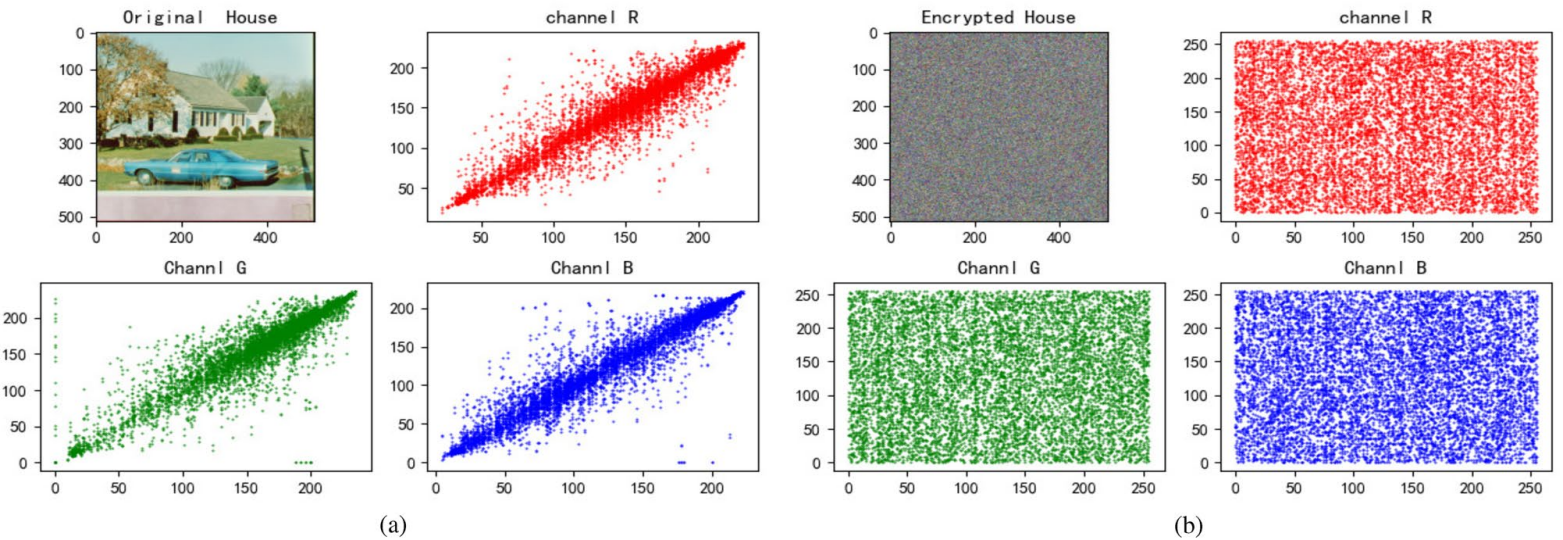
House’s correlation. (**a**) Correlation of House and it’s channels. (**b**) Correlation of encrypted House and it’s channels.

**Figure 11:**
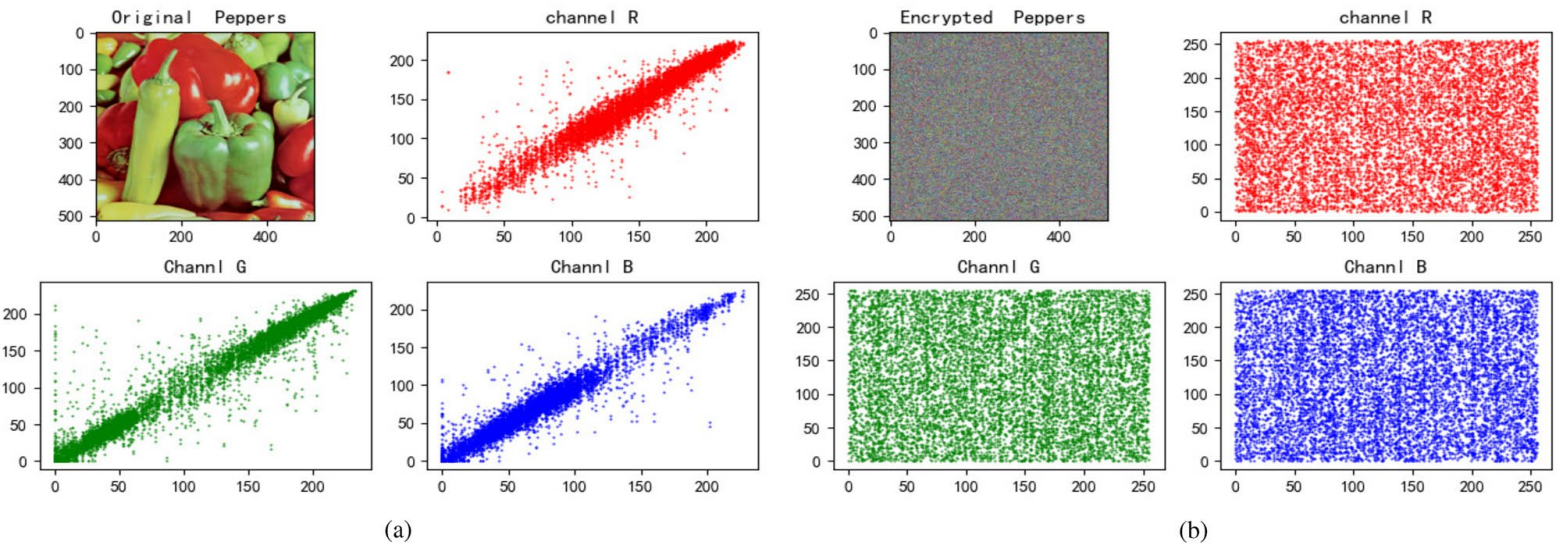
Baboon’s correlation. (**a**) Correlation of Baboon and it’s channels. (**b**) Correlation of encrypted Baboon and it’s channels.

Correlation is a measurement standard for calculating the degree of correlation between two variables. Generally speaking, the degree of correlation between adjacent pixels of the image to be encrypted is high commonly, and a third party can infer the characteristics of the surrounding pixels through a pixel. Therefore, image encryption must decrease the correlation as much as possible. We calculate the correlation of encrypted images with using the formula (17). The value range of the correlation coefficient is [$-1,1$] that the absolute value of the correlation coefficient approaches 0, indicating that the correlation is smaller, and the attack is resisted. The stronger the ability is, the better the effect of image encryption is.

17 $$\begin{aligned} R_{y, x}=\frac{\sum _{i=1}^{W}\left( y_{i}-E(y)\right) \left( x_{i}-E(x)\right) }{W \sqrt{D(y)} \sqrt{D(x)}} \end{aligned}$$

where $D(y)=\frac{1}{W} \sum _{i=1}^{W}\left( y_{i}-E(y)\right) ^{2}$, $E(y)=\frac{1}{W} \sum _{i=1}^{W} y_{i}$. *y* and *x* are the adjacent pixels, and *W* is the total number of pixels in the image. We chose Lena, House and Baboon as the test images to measure the correlation between the original image and the encrypted image of the three images. Firstly, 3000 couples of pixels are selected for each image, and then the correlations in the horizontal, vertical, and diagonal lines are tested respectively. The test results are shown in Figs. 9, 10 and  11 and Table 4. It can be seen that the correlation of the original image is basically linear, while the distribution of the encrypted image is uniform and disorderly. Through the comparison of the two, we can conclude that the encrypted image is weakly correlated and this methods has sound effects.

**Table 4:** Correlation of the original images and encrypted images.

Correlation	Horizontal	Vertical	Diagonal
Original image: Jetplane	0.9685	0.9507	0.9275
Encrypted image: Jetplane	0.0125	0.0134	0.0071
Original image: House	0.9574	0.9620	0.9266
Encrypted image: House	0.0122	0.0098	0.0083
Original image: Baboon	0.9000	0.8349	0.8069
Encrypted image: Baboon	0.0143	0.0103	0.0103

### Key space analysis

For an algorithm of image encryption, it’s trustworthy to have a vast key space. So that external eavesdroppers cannot obtain information through brute force enumeration. The Rubik’s Cube encryption scheme based on chaotic mapping has better security in some real-time confidential communications,however, the key space of traditional chaotic encryption is limited and there is still a risk of being cracked. The encryption scheme based on alternate quantum walk and Rubik’s cube rotation which is put forward in this paper uses the characteristics of alternate quantum walk. While quantum walk are sensitive to the initial state and non-periodic to generate a theoretically infinite space key. Assuming that the initial state of quantum walk is $\left| \psi _{0}\right\rangle$, after the unitary operation of N steps, the final state is $\left| \psi _{N}\right\rangle$:

18 $$\begin{aligned} \left| \psi _{N}\right\rangle ={\hat{U}}^{N}\left| \psi _{0}\right\rangle \end{aligned}$$

The probability of getting a walker at position (y, x) is:

19 $$\begin{aligned} P_{Y, X}=\sum \left| \left\langle y, x_{3} 0\left| {\hat{U}}^{N}\right| \psi _{0}\right\rangle \right| ^{2}+\sum \left| \left\langle y, x, 1\left| {\hat{U}}^{N}\right| \psi _{0}\right\rangle \right| ^{2} \end{aligned}$$

Since the possibility of determining an initial state and decomposing a sum of squares is almost zero, there is endless possibility in the key space. In the conventional computer simulation quantum environment, that is, the precision is $10^{-16}$, the parameters of the quantum random walk consist of four numbers, two of which are obtained by calculating the hash value of the image, and the space size is $2^{256}$, the space size of the other two parameters is $(10^{16})^{2}$, so the key space of the encryption scheme is $2^{256} \times 10^{32}$. Thus, in the case that the initial state cannot be obtained, the randomness and unpredictability of the key sequence make the eavesdropper unable to obtain any information, which effectively prevents the information from being cracked and eavesdropped.

### Key sensitivity analysis

In order to obtain the key sensitivity of the algorithm, we change a parameter of the key for encryption, and test the change pixel rate NPCR and the average change intensity UACI between it and the correct key. The closer the NPCR is to 99.6094% and the UACI to 33.4635%, the stronger the key sensitivity is. The test results of NPCR and UACI are demonstrated in Table 5.

**Table 5:** NPCR and UACI between encrypted images with different key parameters.

Image	NPCR/%	UACI/%
Lena	99.5991	33.4537
House	99.5670	33.3913
Baboon	99.6181	33.4429
Peppers	99.6073	33.4508
Ref^29^	99.5850	28.6210
Ref^46^Baboon	99.61935	*****
Ref^16^	99.765	*****
Ref^48^	99.61	33.47
Ref^49^Baboon	99.6045	33.4457

### Analysis of PSNR

PSNR is used to measure the robustness of encrypted images in the field of image encryption. The smaller the value of PSNR is, the greater the difference between the encrypted image and the original image is. Hence, the encryption effect is better. For the original image ${\mathcal {I}}$ and encrypted image ${{I}_{en}}$. The calculation formula of PSNR is:

20 $$\begin{aligned} P S N R=10 \log _{10} \frac{\left( 2^{n}-1\right) ^{2} a b}{\sum _{i=1}^{a} \sum _{j=1}^{b}({I}_{en}(i, j)-{\mathcal {I}}(i, j))^{2}} \end{aligned}$$

where *a* is the width of image, *b* is the height of the image, and *n* is the number of pixels. We tested the PSNR values of Lena, House, Baboon, and House, and the test results are shown in Table 6 below. The test results indicate that encryption method which put forward has better robustness.

**Table 6:** PSNR of three channels.

PSNR	Jetplane	House	Baboon	Peppers
Channel R	8.206	8.713	8.766	9.127
Channel G	7.915	8.348	9.226	7.65
Channel B	8.006	8.356	8.356	7.648

### Different attack analysis

**Figure 12:**
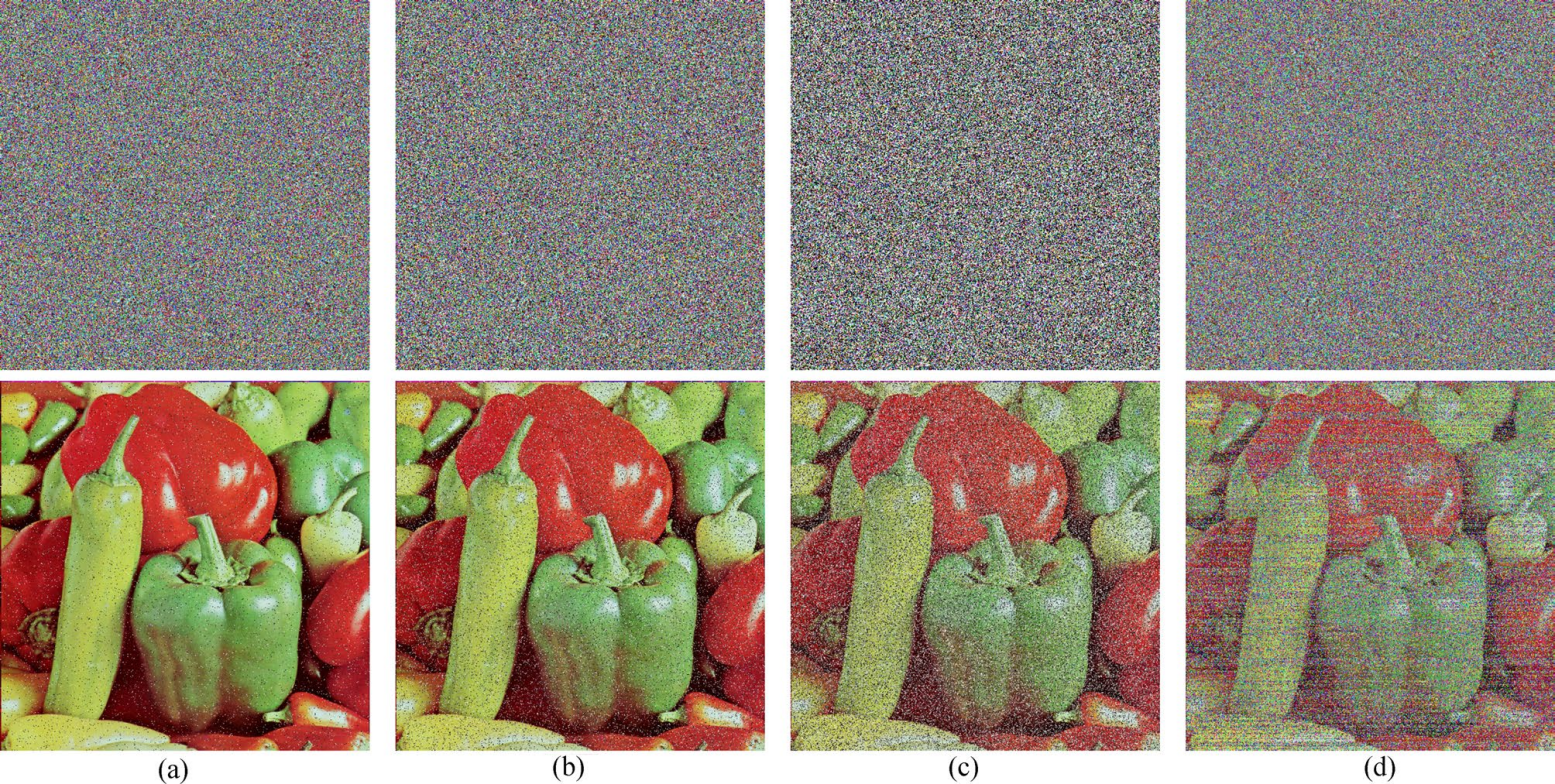
Noise attack. (**a–c**) Pictures which add different degrees of salt and pepper noise, and (**d**) is that adds Gaussian noise.

**Figure 13:**
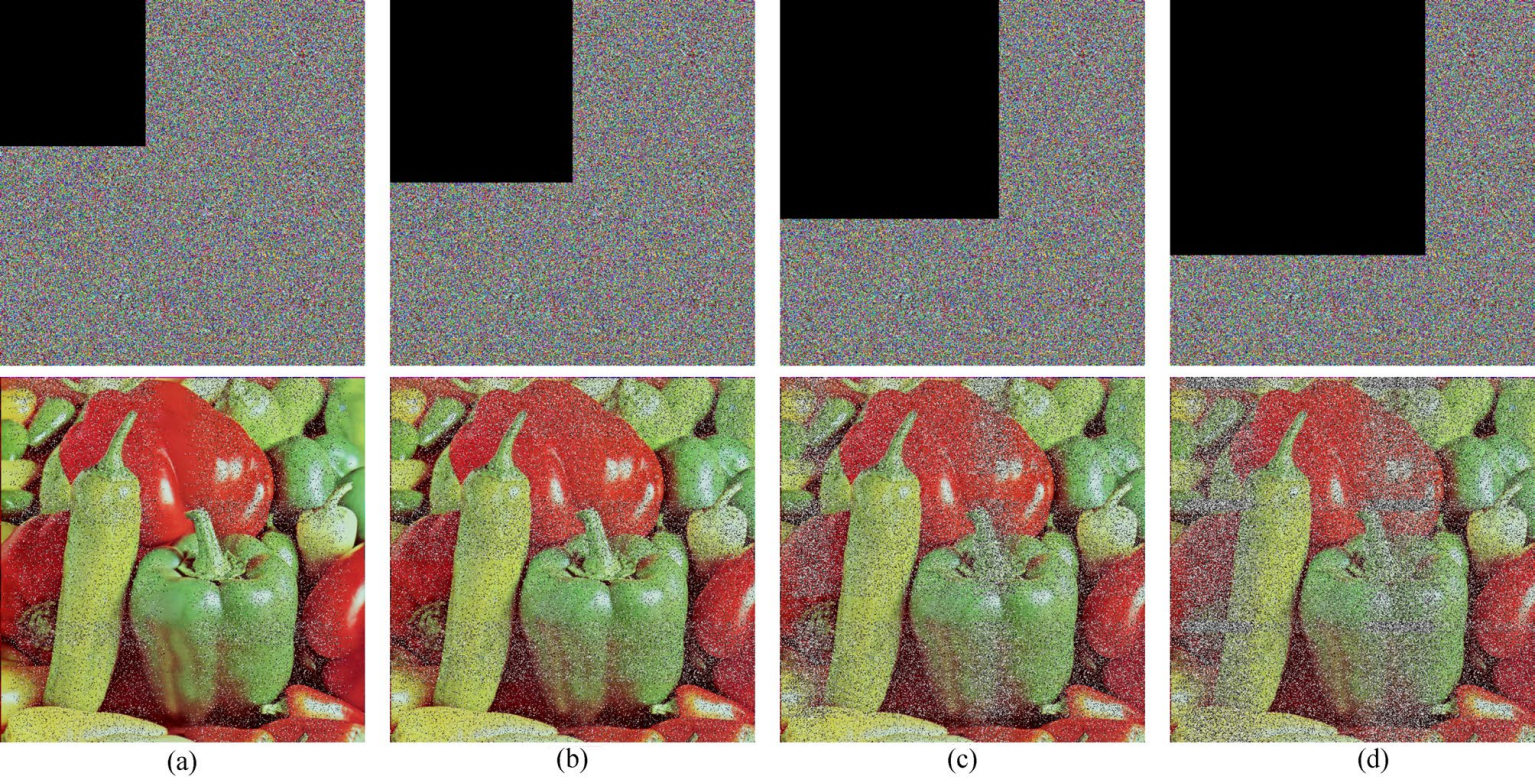
Clipping attack. (**A–D**) The experimental results of 16%, 25%, 36% and 49% of the cut images, respectively.

In the process of network transmission, the transmission of image information will inevitably be affected and destroyed by various factors, resulting in image degradation and pollution, which has a great impact on image decryption. In order to further study and analyze the robustness of the algorithm, this paper validates the common interference and attacks. We add different degrees of salt and pepper noise and Gaussian noise to the image, and the experimental results are shown in Fig. 12; we also crop the image to varying degrees, and the results are shown in Fig. 13. The experimental results show that no matter under the influence of noise or cropping, the algorithm can solve the image with visual information and is robust.

### Differential attack analysis

As a kind of selective plaintext attack, differential attack can effectively test the security of encryption algorithm and the sensitivity of image. By making minor changes to the original plaintext image and analyzing the encrypted image with the unchanged image, the attacker obtains certain relations and rules, and uses them to decrypt other encrypted images to obtain information. Some of the parameters of the quantum random walk of the key generated in this scheme are obtained by calculating the hash value of the image, that is to say, the scheme is sensitive to the image, that is, when the image changes slightly, the encryption result of the image will be very different. In order to confirm this idea, we change one pixel value to the original image, and compare the encrypted image with the encrypted image before the change. The experimental results are shown in the Table 7. Through the data in the table, we find that when the image changes slightly, the encrypted image is very different, especially in the pixel change rate of the two encrypted images. Therefore, the scheme has the ability to resist selective plaintext attack.

**Table 7:** NPCR and UACI between encrypted images.

Image	NPCR/%	UACI/%
Jetplane	99.6116	33.5955
House	99.6134	33.662
Baboon	99.612	33.6651

### Time and space complexity analysis

In this scheme, the pixels of the image are traversed many times in different steps, and the size of the color image is assumed to be $$N*N*3$$. For the algorithm mentioned in the previous chapter, it is considered that the key generated by the alternating quantum random walk and the random sequence are a common part, and the time complexity is $$O(N*N)$$. Then it takes about $$O(N*N*3)$$ to extract the pixel information to construct the Rubik’s cube, and $$O(\frac{1}{6}*9*N*N*3)$$ to complete the scrambling operation for the Rubik’s cube rotation. Finally, the complex time is $$O(N*N*3)$$ after bitwise XOR diffusion processing. Therefore, the time complexity of this scheme is about $$O(11N^2)$$.

The space complexity of the scheme is $O(N ^ 2)$. The step space of alternating quantum random walk to generate key is $O(N*N)$. The space occupied by space scrambling is the space occupied by image segmentation, Rubik’s cube and rotation scrambling and spreading recovery, that is, $O(N*N)+O(1)+O(1)+O(N*N)=O(N*N)$, and the space occupied by diffusion encryption step is $O(N*N)$. To sum up, the space complexity of the scheme is $O(N^2)+O(N^2)=O(N^2)$.

### NIST test analysis

NIST Statistical Test^50^(version NIST SP 800-22 National Institute of Standards and Technology) is suitable for testing the randomness of sequences. The test consists of 15 items that reflect the random performance of the sequence. Table 8 shows the NIST test results of this scheme. It has been mentioned in reference^50^ that when P-Value is greater than or equal to 0.01, this group of data is random, and the result shows that it is passed. The average pass rate of this scheme is 98.9%, and the lowest pass rate is 97%, which is acceptable.

**Table 8:** Result of NIST test for encrypted image.

Statixtical test	P-value	Proportion
Frequence	0.637119	100/100
BlockFrequence	0.401199	100/100
CumulativeSums	0.779188	99/100
CumulativeSums	0.304126	100/100
Runs	0.202268	97/100
LongestRun	0.883171	99/100
Rank	0.040108	99/100
FFT	0.085587	98/100
NonOverlappingTemplate	0.505671	98/100
ApproximateEntropy	–	98/100
Serial	0.779188	99/100
Serial	0.946308	100/100
LinearComplexity	0.816537	99/100
RandomExcursions	0.40783	2/2
RandomExcursionsVariant	0.340435	2/2
Universal	0.449653	2/2

## Summary and prospect

Based on alternate quantum walk and Rubik’s cube transforme, this paper has put forward a novel color image encryption scheme. The core algorithm of this scheme is to generate random sequence through quantum random walk, extract image pixels to form a third-order Rubik’s Cube. Then we control rotation of Rubik’s Cube by using random sequences to realize image scrambling. Through experiments, it is found that proposed scheme has a sound encryption effect. The histogram of encrypted image is evenly distributed, the entropy value is about 7.999, the degree of correlation is low, so it can effectively resist statistical attacks. The algorithm has a vast key space and strong key sensitivity, which can effectively resist brute force attacks. The NPCR of encrypted images is around 99.5978%, and the UACI is around 33.4317%, which can effectively resist differential attacks. The PSNR of the encrypted image is low, and it has better robustness.At present and in the future, we will vigorously promote the combination of quantum walk and classical algorithms, and further apply it to image information encryption in medicine, military and other directions.
